# Determinants of PCR performance (Xpert MTB/RIF), including bacterial load and inhibition, for TB diagnosis using specimens from different body compartments

**DOI:** 10.1038/srep05658

**Published:** 2014-07-11

**Authors:** Grant Theron, Jonny Peter, Greg Calligaro, Richard Meldau, Colleen Hanrahan, Hoosain Khalfey, Brian Matinyenya, Tapuwa Muchinga, Liezel Smith, Shaheen Pandie, Laura Lenders, Vinod Patel, Bongani M. Mayosi, Keertan Dheda

**Affiliations:** 1 Lung Infection and Immunity Unit, Division of Pulmonology & UCT Lung Institute, Department of Medicine, University of Cape Town, Cape Town, South Africa; 2 Johns Hopkins Bloomberg School of Public Health, Department of Epidemiology, Baltimore, MD, USA; 3 Division of Cardiology, Department of Medicine, Groote Schuur Hospital and University of Cape Town, South Africa; 4 Department of Neurology, University of KwaZulu Natal, South Africa; 5 Institute of Infectious Diseases and Molecular Medicine, University of Cape Town, Cape Town, South Africa

## Abstract

The determinants of Xpert MTB/RIF sensitivity, a widely used PCR test for the diagnosis of tuberculosis (TB) are poorly understood. We compared culture time-to-positivity (TTP; a surrogate of bacterial load), MTB/RIF TB-specific and internal positive control (IPC)-specific C_T_ values, and clinical characteristics in patients with suspected TB who provided expectorated (n = 438) or induced sputum (n = 128), tracheal aspirates (n = 71), bronchoalveolar lavage fluid (n = 152), pleural fluid (n = 76), cerebral spinal fluid (CSF; n = 152), pericardial fluid (n = 131), or urine (n = 173) specimens. Median bacterial load (TTP in days) was the strongest associate of MTB/RIF positivity in each fluid. TTP correlated with C_T_ values in pulmonary specimens but not extrapulmonary specimens (Spearman's coefficient 0.5043 versus 0.1437; p = 0.030). Inhibition affected a greater proportion of pulmonary specimens than extrapulmonary specimens (IPC C_T_ > 34: 6% (47/731) versus 1% (4/381; p < 0.0001). Pulmonary specimens had greater load than extrapulmonary specimens [TTPs (interquartile range) of 11 (7–16) versus 22 (18–33.5) days; p < 0.0001]. HIV-infection was associated with a decreased likelihood of MTB/RIF-positivity in pulmonary specimens but an increased likelihood in extrapulmonary specimens. Mycobacterial load, which displays significant variation across different body compartments, is the main determinant of MTB/RIF-positivity rather than PCR inhibition. MTB/RIF C_T_ is a poor surrogate of load in extrapulmonary specimens.

Tuberculosis (TB) is a leading cause of morbidity and mortality, and accurate and rapid diagnostic tests for TB are key to limiting the spread of the epidemic^1^,^2^. In settings with a high HIV prevalence, up to a third of individuals with pulmonary TB may be unable to provide a specimen for testing^3^. Individuals infected with HIV are at an increased risk of developing extrapulmonary TB [which can represent 15–50% of the total TB incidence in HIV prevalent settings^4^,^5^. Due to the paucibacillary but disseminated nature of extrapulmonary TB, and the difficulties associated with specimen acquisition in patients who are sputum scarce, many patients are often difficult to diagnose using conventional techniques and are at risk of increased mortality^6^.

Xpert MTB/RIF (Cepheid, USA) is an automated real-time PCR system that simultaneously detects TB and resistance to rifampicin. The test has excellent accuracy when performed on sputum^7^ and is endorsed by the World Health Organisation (WHO)^8^,^9^ and the USA Federal Drug Administration^10^ for this purpose. In addition to containing PCR reagents and TB-specific primers, each MTB/RIF cartridge contains a set quantity of *Bacillus globigii* spores and a primer pair specific for the DNA in these spores^11^. If the amplification of this internal positive control fails, or occurs after 38 cycles, the test result is designated invalid^12^.

Information regarding Xpert MTB/RIF's performance on non-sputum specimens is emerging^5^,^13^,^14^,^15^,^16^,^17^,^18^,^19^,^20^,^21^,^22^,^23^,^24^,^25^,^26^; however, it is not extensive, nor sufficiently validated in HIV-prevalent settings. MTB/RIF has thus been granted a conditional recommendation for the diagnosis of extrapulmonary TB by the WHO, however, the overall body of evidence has been cited as weak^9^. Furthermore, countries which are presently implementing it for the diagnosis of pulmonary TB, such as South Africa, do not currently permit its routine use on extrapulmonary specimens.

While the relationship between sputum bacillary load (measured using smear microscopy, culture, and MTB/RIF) has been previously characterised^27^,^28^,^29^,^30^,^31^, little is known about the comparative variation in mycobacillary load in fluids from different sites in the body, despite the high burden of extrapulmonary and increased risk of poor outcomes in these patients^32^,^33^. This is critical for informing the development and application of new tests for extrapulmonary TB (where, in some cases, a biomarker-based approach might be optimal). Furthermore, there is no information regarding how the performance of MTB/RIF is influenced by constituents of extrapulmonary specimens or any associated clinical factors. This is important, because salts, proteins or cellular debris are commonly found in non-sputum specimens and can be enriched after specimen processing (e.g., after centrifugation). These can interfere with the amplification enzyme and thereby inhibit the PCR, leading to inaccurate or unreliable results.

In this study, we first compared mycobacterial load in different fluids from different cohorts of patients with TB recruited from similar settings in South Africa (over 1000 patients overall). We identified clinical factors, including HIV co-infection and CD4 count, and specimen characteristics that may modulate liquid culture time-to-positivity (TTP) and MTB/RIF quantitative information [cycle threshold (C_T_) values] in these fluids. We evaluated the degree of MTB/RIF PCR inhibition in each fluid, and how this modified the relationship between MTB/RIF and culture results.

## Methods

### Study information

We have performed a series of studies at the University of Cape Town and the University of KwaZulu-Natal that assessed the accuracy of MTB/RIF for the diagnosis of TB in different body fluids. These were performed in independent cohorts of patients who were clinically suspected of having pulmonary or extrapulmonary TB. Comparative data from these studies for the following specimens types are presented here: expectorated sputum from patients with suspected pulmonary TB attending primary care TB clinics in Cape Town, South African^34^,^35^; induced sputum from sputum-scarce or smear-negative patients attending primary care TB clinics in Cape Town^36^; tracheal aspirates from mechanically-ventilated patients in the intensive care unit of a tertiary level hospital (Groote Schuur Hospital) in Cape Town (#NCT01530568); bronchoalveolar lavage fluid (BALF) from sputum-scarce or smear-negative patients attending the respiratory clinic at the same hospital^23^; pleural fluid from patients with suspected pleural TB attending the same respiratory clinic; cerebral spinal fluid (CSF) from patients with suspected TB meningitis from Inkosi Albert Luthuli Central Hospital in Durban, South Africa^37^,^38^; pericardial fluid from patients suspected of TB pericarditis from four district- and one tertiary-level hospital in South Africa^39^; and urine from patients suspected of TB who are hospitalised in Groote Schuur Hospital^20^. Patients on anti-TB treatment longer than 48 hours were excluded from the analyses. Only patients with paired liquid culture and MTB/RIF results (i.e., from either the same specimen or specimens collected at the same time) were included.

### Ethics statement

Each sub-study was approved by the University of Cape Town or University of Kwa-Zulu Natal research ethics committees, all patients provided written informed consent for participation and the use of their data, and each sub-study was conducted in accordance with the relevant approvals.

### Smear microscopy, liquid culture and Xpert MTB/RIF

When MTB/RIF was performed on sputum, a paired specimen was NALC-NaOH decontaminated, and the sediment used for concentrated fluorescent smear microscopy and liquid culture using the BACTEC MGIT 960 system (BD Diagnostics, USA) performed at a quality-assured accredited reference laboratory. For studies involving other specimen types (induced sputum, tracheal aspirates, BALF, pleural fluid, CSF, pericardial fluid, and urine), the same specimen used for MTB/RIF testing was used for smear microscopy and liquid culture after decontamination. A ~10 ml volume of urine^20^ was first centrifuged and resuspended in 1 ml phosphate buffered saline prior to processing for MTB/RIF. As our study objectives are to compare bacterial load and MTB/RIF-inhibition in different fluids, which would be confounded by different methods of specimen concentration, all other specimens (other than urine) were processed raw and centrifuged, and a volume of 1 ml used. The recommended 2-fold volume of sample buffer was thereafter added and the MTB/RIF procedure started^40^.

### Statistical analyses

Statistical analyses were performed using Graphpad Prism (version 6.0; GraphPad Software, USA, www.graphpad.com), the VassarStats online statistical package (www.vassarstats.net/index.html), and STATA SE (version 12; StataCorp, USA). P-values less than <0.05 were considered significant. A backward elimination strategy was used for multivariate analyses of culture TTP, MTB/RIF *Mycobacterium tuberculosis*-specific C_T_ values, and MTB/RIF inhibition. Variables with p-values <0.100 in univariate analyses were included in the final multivariate model. Fisher's exact test with mid-P correction was used for comparisons between proportions. The Mann-Whitney test to compare medians. Fisher's z transformation was used to compare differences in Spearman's correlation coefficient between TTPs and C_T_ values. For some within-specimen type comparisons of TTP and C_T_ values, there were too few HIV-infected culture-positive patients (n ≤ 5) for meaningful comparisons.

## Results

### Patient characteristics and accuracy of MTB/RIF in different specimen types

Demographic and clinical characteristics are shown in Table 1 for each cohort. The expectorated sputum, induced sputum, tracheal aspirate, BALF, pleural fluid, CSF, pericardial fluid, and urine cohorts had 428, 128, 71, 152, 76, 152, 131, and 173 patients, respectively. The sensitivity and specificity of MTB/RIF for the detection of TB in each specimen type and using liquid culture as a reference standard has been described elsewhere^20^,^23^,^34^,^35^,^41^,^38^ (these studies also examined the impact of centrifugation on MTB/RIF performance), but is also shown in Table 2. The sensitivity of MTB/RIF in pulmonary specimens compared to extrapulmonary specimens was 82% (141/172) versus 50% (48/97; p < 0.0001) and the specificity was 96% (595/617) versus 86% (225/262; p < 0.0001). In contrast, the sensitivity of smear microscopy in pulmonary and extrapulmonary specimens was 60% (73/122) and 2% (2/96), respectively.

### Culture time-to-positivity in different types of specimens

#### Overall

**Figure 1:**
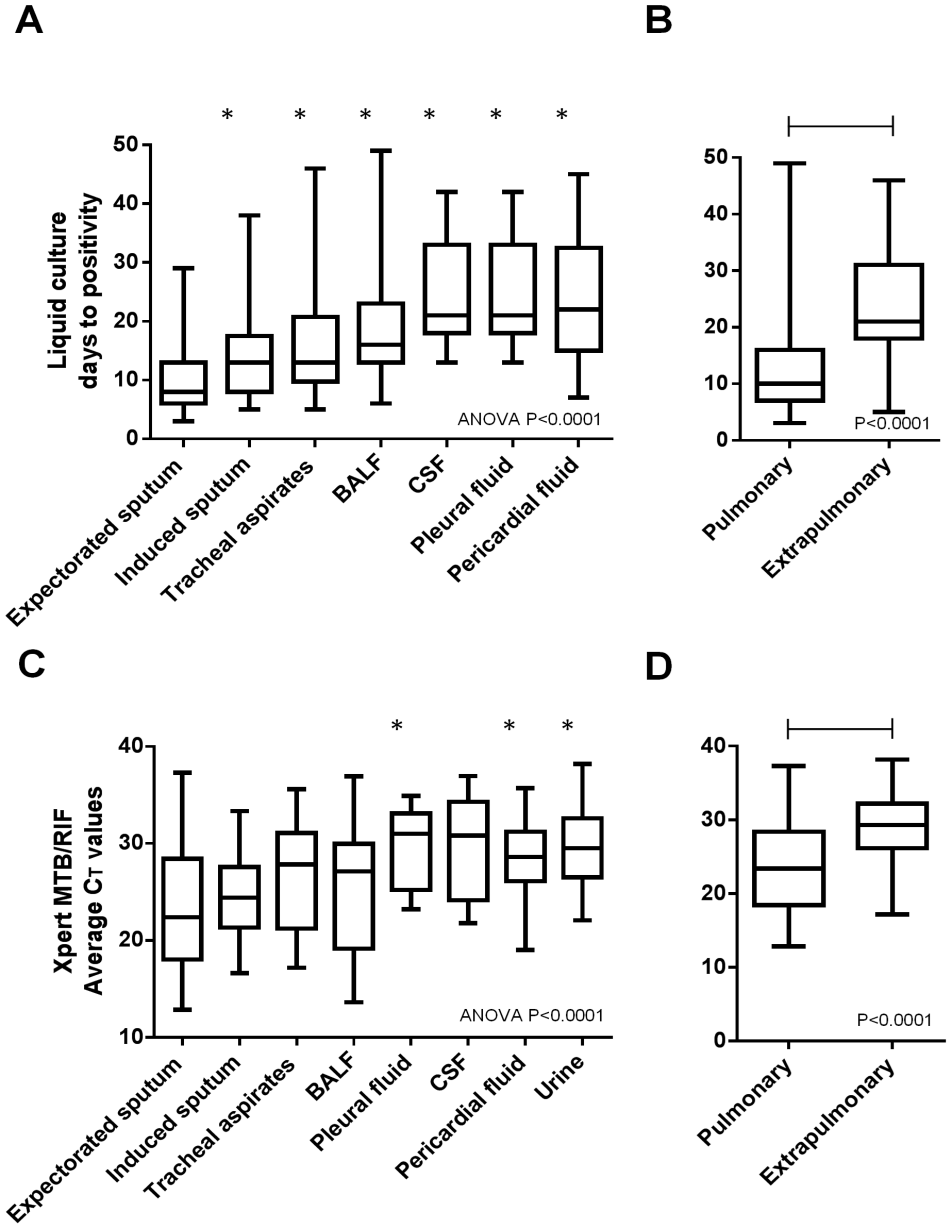
Comparison of culture days-to-positivity (A and B) and Xpert MTB/RIF C_T_ values (C and D) in different fluids, and in pulmonary specimens versus extrapulmonary specimens. Asterisks indicate specimen types with significantly lower bacterial load than expectorated sputum or pulmonary specimens. Abbreviations: BALF, bronchoalveolar lavage fluid; CSF, cerebral spinal fluid. No urine specimens were culture-positive.

Liquid culture TTP was the shortest in expectorated sputum compared to culture-positive specimens of other types (indicating greater bacillary load; ANOVA p < 0.0001), and in pulmonary specimens was shorter than in extrapulmonary specimens [11 (7–16) vs. 22 (18–33.5); p < 0.0001] (Figure 1A and B; Table 2).

#### Differences in TTP in Xpert MTB/RIF-positive and –negative specimens

**Figure 2:**
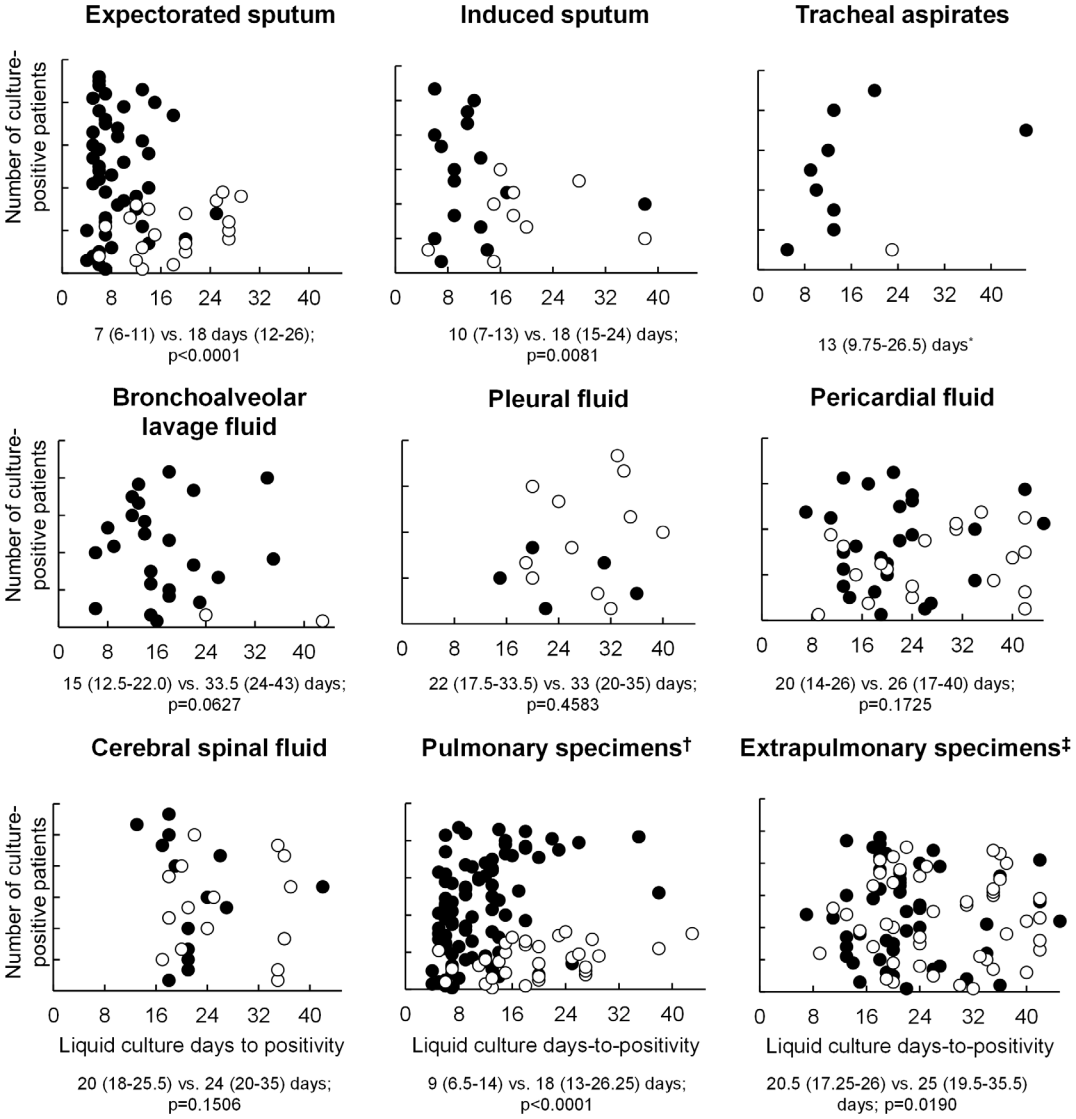
Scatter plots of days-to-positivity in different types of liquid culture-positive specimens obtained from separate patient cohorts. Each circle represents an individual specimen. Solid circles indicate specimens which were Xpert MTB/RIF-positive, whereas empty circles indicate Xpert MTB/RIF-negative specimens. Comparisons below each graph are between median (IQR) TTPs for Xpert-MTB/RIF-positive vs. –negative specimens for that fluid. *Only one MTB/RIF-negative, culture-positive tracheal aspirate specimen was present; Fluids from the lung include expectorated sputum, induced sputum, tracheal aspirates, and bronchoalveolar lavage fluid; Fluids from elsewhere in the body include pleural fluid, cerebral spinal fluid, and pericardial fluid. No patients had culture-positive urine.

Scatter plots of TTP in MTB/RIF-positive and –negative culture specimens of different types are shown in Figure 2. MTB/RIF-positive, culture-positive expectorated sputum and induced sputum both had a shorter TTP compared to those that were MTB/RIF-negative [7 (6–11) vs. 18 days (12–26; p < 0.0001) for expectorated sputum, and 10 (7–13) vs. 18 days (15–24; p = 0.0081) for induced sputum] but not for the other specimen types tested.

#### Differences in TTP according to HIV status

Median TTPs (IQR) amongst culture-positive patients were shorter for expectorated sputum and induced sputum in HIV-uninfected compared to -infected patients [7.5 (6–12) vs. 11.50 (7–15.75) days for expectorated sputum (p = 0.0339); 9 (6.5–12.5) vs. 15.50 (9.75–19.5) days for induced sputum (p = 0.0352)]. When data were pooled, patients that were HIV-uninfected had a similar TTP to those that were HIV-infected [9 (7, 14.25) vs. 13 (7, 18.5) days for pulmonary specimens (p = 0.0726); 25 (18.5, 31.5) vs. 21 (18, 35) days for extrapulmonary specimens (p > 0.9999)].

#### Differences in TTP according to CD4 count

HIV-infected patients with a CD4 count ≤200 cells/μl had a longer median TTP versus those with a CD4 count >200 cells/μl for expectorated sputum [14 (10.25, 20.00) vs. 8 (6, 12) days (p = 0.0027)] and CSF [26 (21, 36) vs. 18.5 (18, 20.25) days (p = 0.0110)], but not for induced sputum [24 (17.5, 36.5) vs. 16.5 (12.75, 24.5) days (p = 0.1071)] or pericardial fluid [21 (15, 27) vs. 24 (17.5, 36.5) days (p = 0.4755)]. When data from HIV-infected patients were pooled, patients with a CD4 count ≤200 cells/μl had a longer median TTP compared to those with a CD4 count >200 cells/μl for pulmonary specimens [15 (11.25, 20) vs. 9.5 (6, 14.5) days (p = 0.0052)], but not for extrapulmonary specimens [22 (19, 35) vs. 20 (18, 24) days (p = 0.2241)].

#### Correlates of time-to-positivity

Multivariable linear regression analyses of culture-positive patients showed the following clinical and demographic factors to be associated with increased TTP: younger age (p = 0.035) and HIV-infection (p = 0.047) for induced sputum (Table S2), previous TB (p = 0.022) for tracheal aspirates (Table S3). No significant associations were found for the other fluids or pooled pulmonary data after multivariable adjustments were performed (see supplement).

### Xpert MTB/RIF-generated cycle threshold values in different types of specimens

#### Overall

When median MTB/RIF-generated cycle threshold values (C_T_ values; a smaller C_T_ value indicates greater load) (IQR) were compared across fluids, those from pleural fluid, CSF, pericardial fluid, and urine were greater than expectrated sputum (Figure 1C and D; Table 2). C_T_ values in pulmonary specimens were lower than in extrapulmonary specimens [23.4 (18.5–28.4) vs. 29.4 (26.4–32.2); p < 0.0001].

#### Differences in C_T_ values according to HIV status

Median C_T_ values (IQR) amongst MTB/RIF-positive patients were similar in HIV-uninfected patients versus -infected patients for expectorated sputum [21.37 (17.71–26.71) vs. 25.09 (18.60–31.08; p = 0.1027)] or induced sputum [24.41 (21.87–29.11) vs. 23.34 (18.37–26.77; p = 0.5363)], and no differences were detected when pooled pulmonary or extrapulmonary data were used [23.43 (18.02–28.33) vs. 24.40 (19.05–30.85) for pulmonary specimens (p = 0.3698); 30.75 (27.65–32.88) vs. 29.00 (26.50–32.04) for extrapulmonary specimens (p = 0.3921)].

#### Differences in C_T_ values according to CD4 count

HIV-infected patients with a CD4 count ≤200 cells/μl had higher C_T_ values versus those with a CD4 count >200 cells/μl for expectorated sputum [29.81 (24.75, 31.95) vs. 20.60 (17.74, 27.30; p = 0.0125)], but not for pericardial fluid [29.15 (26.58, 31.53) vs. 28.10 (25.23, 30.68; p = 0.6390)] or urine [29.53 (26.18, 32.0) vs. 31.01 (28.58, 34.12; p = 0.1532)]. Patients with a CD4 count ≤200 cells/μl had higher median C_T_ values compared to those with a CD4 count >200 cells/μl for pulmonary specimens [28.68 (21.57, 32.25) vs. 20.70 (17.74, 26.36; p = 0.0119)] but not for extrapulmonary specimens [29.47 (26.41, 32.01) vs. 29.51 (25.99, 33.19; p = 0.8033)].

#### Correlates of MTB/RIF-positivity

Multivariable logistic regression analyses showed MTB/RIF-positivity to be associated (p ≤ 0.100) with TTP for expectorated sputum (p < 0.001; Table S1), induced sputum (p = 0.078; Table S2), BALF (p = 0.082; Table S4), and CSF (p = 0.029; Table S6), whereas for pericardial fluid HIV-infection was the only significant associate (p = 0.010; Table S8). Patients who are male or had previously had TB were less likely to have MTB/RIF-positive urine (p-values of 0.051 and 0.054, respectively; Table S10). When pooled pulmonary data were examined, patients who were HIV-infected (p = 0.059) and had a longer TTP (p < 0.001) were less likely to be MTB/RIF-positive (Table S5). Extrapulmonary specimens with a longer TTP (p = 0.003) were also less likely to be MTB/RIF-positive and HIV-infection (p = 0.013) was associated with an increased likelihood of MTB/RIF-positivity (Table S9).

### Comparative PCR inhibition in different specimen types

#### Overall

**Figure 3:**
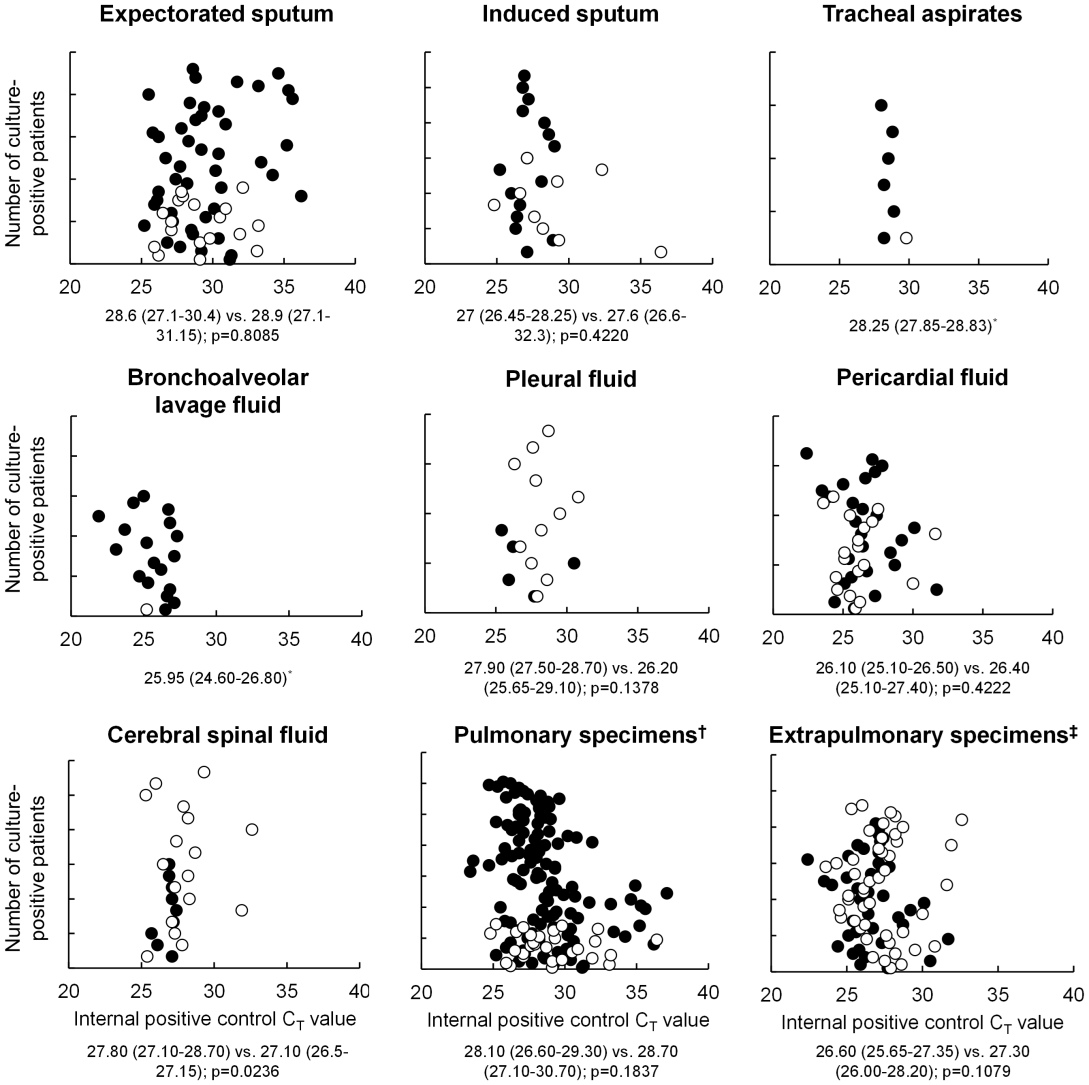
Scatter plots of Xpert MTB/RIF internal positive control (IPC) cycle threshold values in different types of culture-positive specimens obtained from separate patient cohorts. Each circle represents an individual specimen. Solid circles indicate patients who were Xpert MTB/RIF-positive, whereas empty circles indicate Xpert MTB/RIF-negative specimens. Comparisons are between median (IQR) IPC C_T_s for Xpert-MTB/RIF-positive vs. –negative specimens. *Only one MTB/RIF-negative, culture-positive tracheal aspirate specimen and one MTB/RIF-negative, culture-positive BALF specimen were present; Pulmonary specimens include expectorated sputum, induced sputum and bronchoalveolar lavage fluid; Extrapulmonary specimens include tracheal aspirates, pleural fluid, cerebral spinal fluid, and pericardial fluid. No patients had culture-positive urine.

Scatter plots of IPC C_T_ values (smaller IPC C_T_ values indicate less inhibition) are shown in Figure 3. Internal control C_T_ values for expectorated sputum differed to those for induced sputum, tracheal aspirates, BALF, pleural fluid, and CSF, and were similar for pulmonary specimens and extrapulmonary specimens. The proportion of MTB/RIF results with an IPC C_T_ value >34, which have been shown to be due to inhibition in sputum^30^, for expectorated sputum, induced sputum, tracheal aspirates, BALF, pleural fluid, CSF, pericardial fluid, and urine were 9% (39/433), 5% (6/128; p-value compared to expectorated sputum of 0.1140), 3% (2/68; p = 0.0898), 3% (2/76; p = 0.0596), 1% (1/131; p = 0.0013), and 1% (1/142; p = 0.0007), respectively. Collectively, the proportion of MTB/RIF results with an internal control C_T_ value >34 for pulmonary specimens and extrapulmonary specimens was 6% (47/731) and 1% (4/381; p < 0.0001), respectively. Median (IQR) IPC C_T_ values were similar for comparisons between MTB/RIF-positive and –negative culture-positive specimens of each type, except for CSF [27.80 (27.10–28.70) vs. 27.10 (26.5–27.15); p = 0.0236]. MTB/RIF-negative, culture-positive pulmonary specimens and extrapulmonary specimens had median IPC C_T_ values of 28.45 (27.10, 31.15) and 27.30 (26.05, 28.20), respectively (p = 0.0048).

#### Correlates of inhibition

When multivariable linear regression analyses were performed (p ≤ 0.100), female gender was associated with decreased internal control C_T_ values for expectorated sputum (p = 0.007; Table S1), HIV infection for CSF (p = 0.078; Table S6), older age for pericardial fluid (p = 0.089; Table S8), and reduced protein concentration in urine (p = 0.072; Table S10). There was no association between MTB/RIF positivity and internal control C_T_ value for each of the other fluids tested (see supplement), however, when pulmonary data and extrapulmonary data were pooled, female gender (p = 0.008) and younger age (p = 0.076) were respectively associated with less inhibition (Table S4 and S9) for each specimen type respectively.

### Correlation between time-to-positivity and cycle threshold values in different specimen types

**Figure 4:**
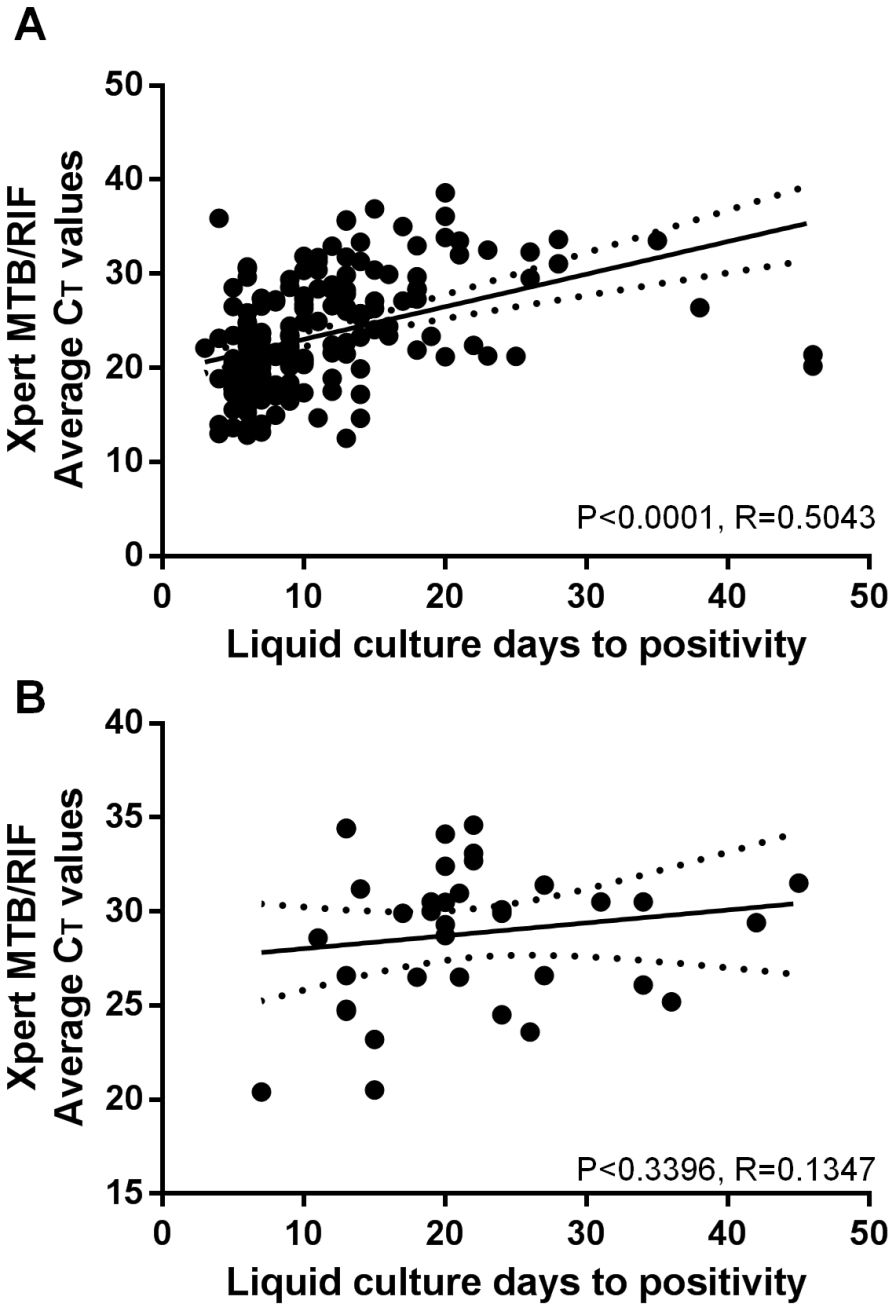
Correlation between liquid culture time-to-positivity and Xpert MTB/RIF cycle threshold values in (A) pulmonary specimens and (B) extrapulmonary specimens.

When the strength of the correlation between culture TTPs and C_T_ values, which may be modulated by PCR inhibition, were compared, similar Spearman correlation coefficients (p-value vs. expectorated sputum) of 0.501, 0.623 (p = 0.5252), 0.100 (p = 0.5287), −0.051 (p = 0.5287), and 0.199 (p = 0.1211) for expectorated sputum, induced sputum, pleural fluid, CSF, and pericardial fluid, respectively were obtained. When data for pulmonary specimens were pooled (Figure 4), TTPs and C_T_ values were correlated (Spearman coefficients of 0.5043; p < 0.0001), however, there was no significant correlation amongst extrapulmonary specimens (Spearman coefficient of 0.1437; p = 0.4032), and the correlation observed amongst pulmonary specimens was stronger (p = 0.030). C_T_ values correlated less strongly with low or high levels of bacterial load, rather than due to any intrinsic properties of extrapulmonary specimens. For example, although C_T_ values and TTP exhibited a significant correlation overall for pulmonary specimens, this was not present amongst specimens with a TTP in the bottom (Spearman coefficient of 0.1805; p = 0.1535) or top tertile (Spearman coefficient of 0.0899; p = 0.5139), but was amongst those in the middle tertile (Spearman coefficient of 0.3758; p = 0.0017).

## Discussion

This study is the first to compare mycobacterial load using culture TTP, MTB/RIF-generated C_T_ values, and MTB/RIF inhibition in specimens from different body compartments. Briefly, our key findings are: (i) compared to expectorated sputum, MTB/RIF is inhibited more in induced sputum, tracheal aspirates, and BALF, but less in pleural fluid; (ii) “false-negative” MTB/RIF results (MTB/RIF-negative, culture-positive) from CSF displayed a greater inhibition compared to “true-positive” results, and pulmonary specimens inhibited MTB/RIF more than extrapulmonary specimens; (iii) C_T_ values correlate with TTP in pulmonary specimens but not in extrapulmonary specimens, suggesting the assay to be unsuitable for estimation of mycobacterial load amongst patients with extrapulmonary TB; (iv) TTP is the strongest correlate of MTB/RIF-positivity in both pulmonary specimens and extrapulmonary specimens, even after adjusting for inhibition; and (v) extrapulmonary specimens are more paucibacillary than pulmonary specimens and, of the pulmonary specimens, expectorated sputum had the highest bacillary load.

We found pulmonary specimens to have a greater proportion of MTB/RIF results with evidence of inhibition [IPC C_T_ value >34^30^] compared to extrapulmonary specimens. It is likely that this is driven by the viscous nature of sputum which, even after the addition of sample buffer, may not be completed homogenised and thus still interefere with the reaciton. Importantly, the inhibitory effect caused by the viscous nature of some sputum specimens is likely offset by the thick mucous within it, which has been shown to contain over 30-fold more bacilli than the watery component, and thus the overall sensitivity remains good^30^.

In our study, we found “false-negative” MTB/RIF results to display more inhibition on CSF than those that are “true-positive”, suggesting that this fluid contains material that interferes significantly with the PCR and thus may be a cause of false-negative results. This is the first description of MTB/RIF inhibition in extrapulmonary specimens. Interestingly, we have shown in a separate study^41^ that, if a 3 ml volume of CSF is centrifuged, the pellet washed, and resuspended in buffer prior to testing, the sensitivity of MTB/RIF improves by almost 40%. In addition to concentrating the bacilli in the specimen, this centrifugation and resuspension step likely also removes PCR inhibitors. Such an approach should be considered for other fluids that inhbit PCR-based tests. The other types of extrapulmonary specimens analysed did not display evidence of significant inhibition.

Several studies have detailed the performance of MTB/RIF on extrapulmonary specimens and pulmonary specimens other than sputum^5^,^13^,^14^,^15^,^16^,^17^,^18^,^19^,^20^,^21^,^22^,^23^,^24^,^25^,^26^,^42^, however, there is little comparative data on how bacilliary load in these differents fluids vary. We found extrapulmonary specimens to have less bacillary load than pulmonary specimens and, in our mulitvariate analyses, bacterial load was the chief determinant of MTB/RIF positivity in both pulmonary specimens and extrapulmonary specimens, rather than inhibition, or any other of the clinical and demographic characterstics examined.

In pulmonary specimens, HIV-infection was associated with a decreased likelihood of a positive MTB/RIF result, however, in extrapulmonary specimens, HIV-infection was associated with an increased likelihood of MTB/RIF-posivity. This is reflective of the lower bacillary load seen in the lungs of HIV-coninfected patients with pulmonary TB (due to the lower frequency of caviation in these patients) compared to those who are HIV-uninfected. In contrast, patients who are HIV-infected displayed a higher TB bacillary load in specimens from extrapulmonary sites than those who were HIV-uninfected, and thus those who are HIV-infected are more likely to be MTB/RIF-positive for EPTB. Although EPTB is more frequent in HIV-infected patients, their extrapulmonary bacillary load is lower than HIV-infected patients with pulmonary TB. This means that EPTB specimens with a concetration of bacilli below the limit of detection of MTB/RIF will occur more frequently, and that patients with suspected TB who have a negative MTB/RIF result should still be investigated further. A further rammification of the low load seen in extrapulmonary specimens is that in fluids such as pleural fluid or pericardial fluid a biomarker-based approach using a molecule such as interferon-γ might be superior^43^ to a nucleic acid amplication assay. Thus, MTB/RIF is not necessarily a “one size fits all”, although it does universally outperform microscopy (the only alternative rapid test in some settings)^7^.

As has been documented by others^23^,^28^,^30^,^34^,^44^,^45^, we found MTB/RIF-generated C_T_ values to correlate significantly with culture time-to-positivity in pulmonary specimens. Such a conclusion is important because, for pulmonary TB, sputum bacillary load at diagnosis is one of the strongest baseline predictors of long-term outcome^46^,^47^,^48^,^49^, and could thus be used for the prognostication of patients. We now show there is no correlation with bacterial load in extrapulmonary specimens, and that this appears to be as a result of the C_T_ values-TTP correlation deteriorating at low levels of bacterial load. While a direct association between baseline MTB/RIF C_T_ values and clinic outcome has not yet been demonstrated, it appears that MTB/RIF would not be useful for such a purpose amongst patients with extrapulmonary TB, or for a meaningful estimation of disease severity as approximated by bacterial load.

This study has limitations. Although this is the first study to report on MTB/RIF inhibition in fluids other than sputum, we did not capture data on specimen-specific factors such as viscosity, appearance, or salt concentration, which may interefere with MTB/RIF. Although shown to be useful by us and other^25^,^41^,^42^, we did not assess bacterial load and inhibition in centrifuged specimens (other than urine), as these data were not available for all the specimen types included in this study and, when it were, different specimen volumes were used for concentration. Our analyses were also restricted in some instances by the comparatively small number of culture-positive specimens, especially after stratification by MTB/RIF- and/or HIV-status and specimen type; however, the size of the cohort in each of these parent studies is mostly in excess of that reported elsewhere^7^. While we^28^,^31^ and others^30^ have described how MTB/RIF can be used to predict smear-positivty in sputum, we were unable to replicate such an analysis here, due to the small number of non-sputum specimens that were smear-positive. Our specimens of different types were stored for different durations, and this may have influenced some differences, however, recent work has demonstrated that MTB/RIF accuracy is not significantly affected by storage duration^7^,^18^, suggesting this effect, if any, to be minimal.

In summary, this study has demonstrated that low mycobacillary load in extrapulmonary specimens is, rather than inhibition, primarily responsible for the diminished sensitivity of MTB/RIF in these specimens compared to those from the pulmonary system. While “false-negative” CSF displayed more inhibition than “true-positive” specimens, pulmonary specimens displayed the most inhibition overall, suggesting that MTB/RIF quantitative information might not be useful in a significant minority of patients with suspected pulmonary TB. Furthermore, the quantitative information generated by MTB/RIF from extrapulmonary specimens does not correlate with bacterial load, and is unlikely to be useful. Future studies on the exact clinical and specimen-specific determinants of MTB/RIF inhibition are important, as well additional specimen preparation steps that may reduce inhibition, especially if MTB/RIF quantitative information will used for patient management.

## Supplementary Material

Supplementary Information Online supplement

## Figures and Tables

**Table 1:** Demographic and clinical characteristics of the different cohorts. Only significant p-values for comparisons between expectorated sputum and other specimen types are shown. P-values for pulmonary specimens and extrapulmonary specimens are for comparisons between the two groups. For protein concentration, only significant p-values for comparisons versus pleural fluid are shown. *Two patients in the urine group were missing age information. The following number of patients in each group were of unknown smoking status: 12 in the expectorated sputum group, 6 in the tracheal aspirate group, 10 in the BALF group, and 5 in the pleural fluid group. The following number of patients in each group refused HIV testing or had missing data: 31 in the expectorated sputum group, 3 in the induced sputum group, 11 in the tracheal aspirate group, 26 in the BALF group, 23 in the pleural fluid group, and 6 in the pericardial fluid group. HIV-infection was an eligibility criterion for the parent study of this cohort. **The following number of patients infected with HIV in each group were missing CD4 count information: 6 in the expectorated sputum group, 1 in the induced sputum group, 1 in the tracheal aspirate group, 1 in the BALF group, 9 in the CSF group, 4 in the pericardial fluid group, and 9 in the urine group. The following number of patients in each group were missing information about their previous TB: 10 in the expectorated sputum group, 4 in the tracheal aspirate group, 6 in the BALF group, 7 in the pleural fluid group, and 4 in the pericardial fluid group. Abbreviations: BALF, bronchoalveolar lavage fluid; CSF, cerebral spinal fluid; IQR, interquartile range; ND, not done

Specimen type	Expectorated sputum (n = 438)	Induced sputum (n = 128)	Tracheal aspirates (n = 71)	BALF (n = 152)	Pleural fluid (n = 76)	CSF (n = 152)	Pericardial fluid (n = 131)	Urine (n = 173)	Pulmonary specimens (n = 789)	Extra-pulmonary specimens (n = 532)
**Demographic characteristics**										
Median age in years (IQR)*	39 (30–49)	39 (30–59)	36 (27–49)	46 (33–55) p = 0.002	55 (38–65) p < 0.0001	32 (26–37) p < 0.0001	35 (29–42)	35 (29–40) p < 0.01	39 (31–50)	35 (28–42) p < 0.0001
Male gender (%)	298 (67)	63 (49) p < 0.0001	41 (58)	82 (54) p = 0.002	45 (58)	57 (38) p < 0.0001	82 (63)	69 (40) p < 0.0001	61 (481/789)	48 (253/532) p < 0.0001
Tobacco smoker (%)	258 (61)	52 (41) p < 0.0001	21 (32) p < 0.0001	41 (29) p < 0.0001	19 (27) p < 0.0001	NR	NR	35 (20) p < 0.0001	49 (372/761)	22 (54/244) p < 0.0001
**Clinical characteristics**										
HIV-infected (%)	128 (31)	47 (38)	25 (42)	23 (18) p = 0.004	9 (17) p = 0.030	131 (86) p < 0.0001	90 (72) p < 0.0001	N/A	32 (223/718)	80 (403/503) p < 0.0001
Median CD4 count (cells/μl) (IQR) if HIV-infected**	215 (127–360)	250 (148–373)	159 (58–379)	243 (80–451)	102 (68–271)	138 (60–247) p = 0.008	141 (82–256)	84 (45–197) p < 0.0001	211 (122–376)	120 (58–231) p < 0.0001
Previous TB (%)	173 (40)	49 (38)	24 (36)	50 (34)	9 (13) p < 0.0001	41 (27) p = 0.003	40 (31)	62 (47)	38 (296/769)	29 (152/521) p = 0.0006

**Table 2:** Culture time-to-positivity, Xpert MTB/RIF accuracy and cycle threshold values in different fluids. Only significant p-values for comparisons between expectorated sputum and other specimen types are shown. P-values for pulmonary specimens and extrapulmonary specimens are for comparisons between the two groups. *No patients had culture-positive urine. Sensitivity and specificity calculations used liquid culture from either the same or a paired specimen of the same type as a reference standard. 196 patients in the expectorated sputum group and all of the patients in the tracheal aspirate group did not have a smear microscopy result, as this test was not part of the original trial designs. 1 patient in the BALF group was missing a smear microscopy result. Abbreviations: BALF, bronchoalveolar lavage fluid; CSF, cerebral spinal fluid; IQR, interquartile range; C_T_ values, cycle threshold values; IPC, internal positive control

Specimen type	Expectorated sputum (n = 438)	Induced sputum (n = 128)	Tracheal aspirates (n = 71)	BALF (n = 152)	Pleural fluid (n = 76)	CSF (n = 152)	Pericardial fluid (n = 131)	Urine (n = 173)	Pulmonary specimens (n = 789)	Extra-pulmonary specimens (n = 532)
**Liquid culture**										
Percentage culture-positive	25 (109/438)	20 (25/128) p = 0.2100	15 (11/71) p = 0.0837	18 (27/152) p = 0.0724	21 (16/76) p = 0.4721	23 (35/152) p = 0.6456	35 (46/131) p = 0.0210	0	22 (172/789)	18 (97/532) p = 0.1144
Time-to-positivity (days)	8 (6–13)	13 (8–18) p = 0.0251	13 (8–21) p = 0.0489	16 (13–23) p < 0.0001	28 (20–34) p < 0.0001	21 (18–33) p < 0.0001	22 (15–32) p < 0.0001	N/A*	11 (7–16)	22 (18–33.5) p < 0.0001
**Smear microscopy**										
Sensitivity (%)	69 (49/71)	36 (9/25) p = 0.0037	ND	58 (15/26) p = 0.2972	0 (0/15) p < 0.0001	3 (1/35) p < 0.0001	2 (1/46) p < 0.0001	N/A*	60 (73/122)	2 (2/96) p < 0.0001
Specificity (%)	99 (170/171)	100 (103/103) p = 0.4368	ND	99 (124/125) p = 0.8234	100 (59/59) p = 0.5561	100 (117/117) p = 0.4073	100 (85/85) p = 0.4799	100 (173/173) p = 0.3138	99 (397/399)	100 (434/434) p = 0.1398
**Xpert MTB/RIF**										
Sensitivity (%)	83 (90/109)	64 (16/25) p = 0.0394	91 (10/11) p = 0.4793	93 (25/27) p = 0.1969	31 (5/16) p < 0.0001	46 (16/35) p < 0.0001	59 (27/46) p = 0.0016	N/A*	82 (141/172)	50 (48/97) p < 0.0001
Specificity (%)	97 (318/329)	96 (99/103) p = 0.7939	97 (58/60) p = 0.9968	96 (120/125) p = 0.7347	90 (54/60) p = 0.0204	94 (110/117) p = 0.2128	72 (61/85) p < 0.0001	82 (141/173) p < 0.0001	96 (595/617)	86 (225/262) p < 0.0001
Median C_T_ values (IQR)	22.4 (18.1–28.4)	24.4 (21.3–27.6) p = 0.3570	27.9 (21.2–31.1) p = 0.2293	27.1 (19.2–30.0) p = 0.3440	31.0 (25.2–33.1) p = 0.0007)	30.8 (24.1–34.3) p = 0.0016	28.6 (26.1–31.2) p < 0.0001	29.5 (26.5–32.6) p < 0.0001	23.4 (18.5–28.4)	29.4 (26.4–32.2) p < 0.0001
Median IPC C_T_ values (IQR)	26.2 (25–28)	28.1 (26.83–29.10) p < 0.0001	28.2 (27.1–28.85) p = 0.0156	25.55 (24.70–26.93) p = 0.0060	27.6 (26.2–29.4) p < 0.0001	27.15 (26.38–27.98) p = 0.3502	26.50 (25.15–27.6) p = 0.6801	28.20 (27.10–28.85) p = 0.0567	26.50 (25.10–28.20)	26.60 (25.60–28.10) p = 0.2033
