# Supplementary material for: Determinants of PCR performance (Xpert MTB/RIF), including bacterial load and inhibition, for TB diagnosis using specimens from different body compartments
Source: Sci Rep. 2014 Jul 11;4:5658. doi: 10.1038/srep05658 (PMC5375978; doi:10.1038/srep05658)
Supplement: Supplementary Information — Online supplement [file srep05658-s1.doc]

**Determinants of PCR performance (Xpert MTB/RIF), including bacterial load and inhibition, for TB diagnosis using specimens from different body compartments**

Grant Theron^1^, Jonny Peter^1^, Greg Calligaro^1^, Richard Meldau^1^, Colleen Hanrahan^2^, Hoosain Khalfey^1^, Brian Matinyenya^1^, Tapuwa Muchinga^1^, Liezel Smith^1^, Shaheen Pandie^3^, Laura Lenders^1^, Vinod Patel^4^, Bongani M. Mayosi^3^, Keertan Dheda^1, 5*^

^1^Lung Infection and Immunity Unit, Division of Pulmonology & UCT Lung Institute, Department of Medicine, University of Cape Town, Cape Town, South Africa.

^2^Johns Hopkins Bloomberg School of Public Health, Department of Epidemiology, Baltimore, MD USA.

^3^Division of Cardiology, Department of Medicine, Groote Schuur Hospital and University of Cape Town, South Africa.

^4^Department of Neurology, University of KwaZulu Natal, South Africa.

^5^Institute of Infectious Diseases and Molecular Medicine, University of Cape Town, Cape Town, South Africa.

^*^Corresponding author. [Keertan.dheda@uct.ac.za](mailto:Keertan.dheda@uct.ac.za)

**Supplement**

Table S1. Multivariate models for liquid culture time-to-positivity, Xpert MTB/RIF-positivity and IPC C_T_ values in expectorated sputum.

| Characteristic | Outcome: time-to-positivity in culture-positive patients | | | | Outcome: MTB/RIF-positivity in culture-positive patients | | | | Outcome: internal positive control C_T_ value in culture-positive patients | | | |
| --- | --- | --- | --- | --- | --- | --- | --- | --- | --- | --- | --- | --- |
|  | Univariate | | Multivariate | | Univariate | | Multivariate | | Univariate | | Multivariate | |
|  | Coefficient (95% CI) | P-value | Coefficient (95% CI)) | P-value | OR (95% CI) | P-value | OR (95% CI) | P-value | Coefficient (95% CI) | P-value | Coefficient (95% CI) | P-value |
| Age | -0.02 (-0.13, 0.09) | 0.698 | - | - | 1.01 (0.97, 1.06) | 0.446 | - | - | 0.00 (-0.06, 0.05) | 0.880 | - | - |
| Male gender | -0.94 (-3.57, 1.69) | 0.481 | - | - | 2.00 (0.72, 5.57) | 0.184 | - | - | 1.61 (0.38, 2.83) | 0.011 | 1.04 (0.28, 1.79) | 0.007 |
| Smoker | -0.25 (-2.77, 2.25) | 0.839 | - | - | 1.31 (0.48, 3.56) | 0.594 | - | - | 0.30 (-1.1, 1.2) | 0.954 | - | - |
| Previous TB | 1.90 (-0.62, 4.48) | 0.136 | - | - | 1.50 (0.50, 4.54) | 0.477 | - | - | 0.20 (-1.01, 1.41) | 0.744 | - | - |
| HIV-infected | 2.45 (-0.07, 4.97) | 0.057 | - | - | 0.43 (0.15, 1.20) | 0.107 | 0.57 (0.17, 1.92) | 0.360 | -1.16 (-2.38, 0.05) | 0.060 | -0.48 (-1.22, 0.26) | 0.204 |
| Chest radiograph compatible with active TB | -0.23 (-5.16, 4.70) | 0.926 | - | - | 2.65 (0.44, 16.02) | 0.287 | - | - | 1.60 (-0.83, 4.03) | 0.194 | - | - |
| MTB/RIF internal positive control C_T_ value | -0.04 (-0.46, 0.37) | 0.832 | - | - | 1.00 (0.84, 1.19) | 0.984 | - | - | N/A | N/A | N/A | N/A |
| Time-to-positivity (days) | N/A | N/A | N/A | N/A | 0.82 (0.75, 0.90) | <0.001 | 0.83 (0.75, 0.90) | <0.001 | -0.01 (-0.10, 0.02) | 0.832 | - | - |

Table S2. Multivariate models for liquid culture time-to-positivity, Xpert MTB/RIF-positivity and IPC C_T_ values in induced sputum.

| Characteristic | Outcome: time-to-positivity in culture-positive patients | | | | Outcome: MTB/RIF-positivity in culture-positive patients | | | | Outcome: internal positive control C_T_ value in culture-positive patients | | | |
| --- | --- | --- | --- | --- | --- | --- | --- | --- | --- | --- | --- | --- |
|  | Univariate | | Multivariate | | Univariate | | Multivariate | | Univariate | | Multivariate | |
|  | Coefficient (95% CI) | P-value | Coefficient (95% CI) | Coefficient (95% CI) | OR (95% CI) | P-value | OR (95% CI) | P-value | Coefficient (95% CI) | P-value | Coefficient (95% CI) | P-value |
| Age | -0.40 (-0.76, -0.03) | 0.035 | - | - | 1.00 (0.92, 1.09) | 1.000 | - | - | -0.02 (-0.13, 0.09) | 0.709 | - | - |
| Male gender | -0.86 (-8.68, 6.95) | 0.822 | - | - | 0.56 (0.11, 3.07) | 0.511 | - | - | 0.32 (-1.93, 2.57) | 0.769 | - | - |
| Smoker | -1.21 (-9.01, 6.60) | 0.752 | - | - | 0.57 (0.11, 3.07) | 0.511 | - | - | 0.07 (-2.18, 2.33) | 0.947 | - | - |
| Previous TB | -0.80 (-9.59, 7.99) | 0.853 | - | - | 0.46 (0.07, 2.99) | 0.418 | - | - | -0.14 (-2.74. 2.46) | 0.910 | - | - |
| HIV-infected | 7.28 (0.11. 14.45) | 0.047 | - | - | 0.125 (0.01, 1.24) | 0.125 | - | - | 1.14 (-0.99, 3.28) | 0.278 | - | - |
| Chest radiograph compatible with active TB | -1.91 (-10.34, 6.52) | 0.644 | - | - | 1.38 (0.23, 8.30) | 0.728 | - | - | -0.46 (-2.87, 1.95) | 0.693 | - | - |
| MTB/RIF internal positive control C_T_ value | 0.53 (-1.15, 2.20) | 0.520 | - | - | 0.72 (0.45, 1.14) | 0.162 | - | - | N/A | N/A | N/A | N/A |
| Time-to-positivity (days) | N/A | N/A | N/A | N/A | 0.90 (0.79, 1.01) | 0.078 | - | - | 0.04 (-0.08, 0.16) | 0.520 | - | - |

Table S3. Multivariate models for liquid culture time-to-positivity, Xpert MTB/RIF-positivity and IPC C_T_ values in tracheal aspirates.

| Characteristic | Outcome: time-to-positivity in culture-positive patients | | | | Outcome: MTB/RIF-positivity in culture-positive patients | | | | Outcome: internal positive control C_T_ value in culture-positive patients | | | |
| --- | --- | --- | --- | --- | --- | --- | --- | --- | --- | --- | --- | --- |
|  | Univariate | | Multivariate | | Univariate | | Multivariate | | Univariate | | Multivariate | |
|  | Coefficient (95% CI) | P-value | Coefficient (95% CI) | Coefficient (95% CI) | OR (95% CI) | P-value | OR (95% CI) | P-value | Coefficient (95% CI) | P-value | Coefficient (95% CI) | P-value |
| Age | -0.22 (-0.92, 0.49) | 0.505 | - | - | - | - | - | - | 0.03 (-0.07, 0.12) | 0.476 | - | - |
| Male gender | -0.86 (-8.68, 6.95) | 0.822 | - | - | - | - | - | - | -0.25 (-1.75, 1.24) | 0.675 | - | - |
| Smoker | 10.55 (-7.87, 28.97) | 0.218 | - | - | - | - | - | - | -0.07 (-1.57, 1.42) | 0.896 | - | - |
| Previous TB | 20.71 (3.93, 37.49) | 0.022 | - | - | - | - | - | - | 0.02 (-2.14, 2.17) | 0.985 | - | - |
| HIV-infected | -10.9 (-35.17, 13.37) | 0.323 | - | - | - | - | - | - | -0.37 (-2.34, 1.61) | 0.596 | - | - |
| Chest radiograph compatible with active TB | -6.8 (-26.61, 13.01) | 0.457 | - | - | - | - | - | - | -0.02 (-1.69, 1.64) | 0.977 | - | - |
| MTB/RIF internal positive control C_T_ value | -2.41 (-23.37, 18.55) | 0.780 | - | - | - | - | - | - | N/A | N/A | N/A | N/A |
| Time-to-positivity (days) | N/A | N/A | N/A | N/A | 0.98 (0.85, 1.12) | 0.765 | - | - | -0.01 (-0.07, 0.05) | 0.780 | - | - |

^*^For age, gender, smoking status, previous TB status, HIV status, and chest radiograph status, there were too few observations to perform regression analyses for MTB/RIF-positivity.

Table S4. Multivariate models for liquid culture time-to-positivity, Xpert MTB/RIF-positivity and IPC C_T_ values in bronchoalveolar lavage fluid.

| Characteristic | Outcome: time-to-positivity in culture-positive patients | | | | Outcome: MTB/RIF-positivity in culture-positive patients | | | | Outcome: internal positive control C_T_ value in culture-positive patients | | | |
| --- | --- | --- | --- | --- | --- | --- | --- | --- | --- | --- | --- | --- |
|  | Univariate | | Multivariate | | Univariate | | Multivariate | | Univariate | | Multivariate | |
|  | Coefficient (95% CI) | P-value | Coefficient (95% CI) | Coefficient (95% CI) | OR (95% CI) | P-value | OR (95% CI) | P-value | Coefficient (95% CI) | P-value | Coefficient (95% CI) | P-value |
| Age | 0.00 (-0.28, 0.27) | 0.978 | - | - | 0.98 (0.90, 1.08) | 0.725 | - | - | -0.01 (-0.06, 0.03) | 0.607 | - | - |
| Male gender | -0.59 (-9.17, 7.99) | 0.888 | - | - | - | - | - | - | 0.98 (-0.48, 2.44) | 0.175 | - | - |
| Smoker | 6.15 (-4.69. 16.99) | 0.252 | - | - | - | - | - | - | 0.51 (-1.92, 2.94) | 0.665 | - | - |
| Previous TB | -5.98 (-16.36, 4.4.) | 0.247 | - | - | - | - | - | - | -0.87 (-2.56. 0.81) | 0.294 | - | - |
| HIV-infected | -1.97 (-17.44, 13.50) | 0.791 | - | - | - | - | - | - | 0.65 (-2.43, 3.73) | 0.645 | - | - |
| MTB/RIF internal positive control C_T_ value | -0.89 (-4.05, 2.28) | 0.562 | - | - | 1.17 (0.32, 4.21) | 0.813 | - | - | N/A | N/A | N/A | N/A |
| Time-to-positivity (days) | N/A | N/A | N/A | N/A | 0.89 (0.78, 1.01) | 0.082 | - | - | -0.02 (-0.10, 0.06) | 0.562 | - | - |

Footnotes: The following information was unavailable for patients in this cohort: chest radiograph status, smoking status. ^*^For age, gender, previous TB status, and HIV status, there were too few observations to perform regression analyses for correlates of MTB/RIF-positivity.

Table S5. Multivariate models for liquid culture time-to-positivity, Xpert MTB/RIF-positivity and IPC C_T_ values in pulmonary specimens (expectorated sputum, induced sputum, tracheal aspirates, and BALF).

| Characteristic | Outcome: time-to-positivity in culture-positive patients | | | | Outcome: MTB/RIF-positivity in culture-positive patients | | | | Outcome: internal positive control C_T_ value in culture-positive patients | | | |
| --- | --- | --- | --- | --- | --- | --- | --- | --- | --- | --- | --- | --- |
|  | Univariate | | Multivariate | | Univariate | | Multivariate | | Univariate | | Multivariate | |
|  | Coefficient (95% CI) | P-value | Coefficient (95% CI) | Coefficient (95% CI) | OR (95% CI) | P-value | OR (95% CI) | P-value | Coefficient (95% CI) | P-value | Coefficient (95% CI) | P-value |
| Age | -0.06 (-0.17, 0.05) | 0.282 | - | - | 0.00 (-0.03, 0.03) | 0.985 | - | - | -0.02 (-0.06, 0.02) | 0.379 | - | - |
| Male gender | -1.76 (-4.42, 0.91) | 0.195 | - | - | 0.41 (-0.37, 1.19) | 0.301 | - | - | 1.77 (0.87. 2.68) | <0.001 | 1.22 (0.32, 2.12) | 0.008 |
| Previous TB | 0.95 (-1.95, 3.84) | 0.521 | - | - | 0.22 (-0.66, 1.10) | 0.627 | - | - | 0.51 (-0.52, 1.54) | 0.327 | - | - |
| HIV-infected | 1.300 (-1.48, 4.08) | 0.358 | - | - | -1.32 (-2.17, -0.47) | 0.002 | -0.94 (-1.19, 0.37) | 0.059 | -0.52 (-1.55, 0.50) | 0.316 | - | - |
| MTB/RIF internal positive control C_T_ value | -0.34 (-0.78, 0.08) | 0.114 | - | - | -0.07 (-0.20, 0.07) | 0.336 | - | - | N/A | N/A | N/A | N/A |
| Time-to-positivity (days) | N/A | N/A | N/A | N/A | -0.09 (-0.12, -0.04) | <0.001 | -0.13 (-0.19, -0.07) | <0.001 | -0.05 (-0.11, 0.01) | 0.114 | - | - |
| Specimen type (compared to expectorated sputum) | | | | | | | | | | | | |
| Induced sputum | 3.61 (0.07, 7.14) | 0.046 | - | - | -0.98 (-1.93, -0.03) | 0.044 | -0.54 (-1.66, 0.59) | 0.350 | -1.37 (-2.59, -1.16) | 0.027 | -0.93 (-2.16, 0.31) | 0.140 |
| Tracheal aspirates | 8.26 (3.21, 13.30) | 0.001 | - | - | 0.75 (-1.37, 2.86) | 0.489 | 2.69 (-0.23, 5.61) | 0.071 | -0.64 (-2.70, 1.41) | 0.539 | -0.47 (-2.16, 0.31) | 0.649 |
| BALF | 8.24 (4.81, 11.66) | <0.001 | - | - | 0.97 (-0.55, 2.50) | 0.212 | 2.73 (0.18, 5.28) | 0.036 | -3.59 (-4.90, -2.28) | <0.001 | -3.17 (-4.49, 01.84) | <0.001 |

Table S6. Multivariate models for liquid culture time-to-positivity, Xpert MTB/RIF-positivity and IPC C_T_ values in cerebral spinal fluid.

| Characteristic | Outcome: time-to-positivity in culture-positive patients | | | | Outcome: MTB/RIF-positivity in culture-positive patients | | | | Outcome: internal positive control C_T_ value in culture-positive patients | | | |
| --- | --- | --- | --- | --- | --- | --- | --- | --- | --- | --- | --- | --- |
|  | Univariate | | Multivariate | | Univariate | | Multivariate | | Univariate | | Multivariate | |
|  | Coefficient (95% CI) | P-value | Coefficient (95% CI) | Coefficient (95% CI) | OR (95% CI) | P-value | OR (95% CI) | P-value | Coefficient (95% CI) | P-value | Coefficient (95% CI) | P-value |
| Age | -0.31 (-0.75, 0.13) | 0.163 | - | - | 1.04 (0.95, 1.44) | 0.370 | - | - | -.04 (-0.15, 0.07) | 0.439 | - | - |
| Male gender | -4.33 (-10.97, 2.30) | 0.193 | - | - | 0.88 (0.22, 3.34) | 0.845 | - | - | -0.38 (-2.02. 1.26) | 0.636 | - | - |
| Previous TB | 5.00 (-2.83. 12/83) | 0.203 | - | - | 1.08 (0.22, 5.21) | 0.927 | - | - | -0.87 (-3.41, 1.75) | 0.499 | - | - |
| HIV-infected | 6.72 (-5.08, 18.52) | 0.255 | - | - | - | - | - | - | -1.87 (-3.96, 0.23) | 0.078 | - | - |
| MTB/RIF internal positive control C_T_ value | 0.28 (-2.46, 3.02) | 0.835 | - | - | 0.47 (0.17, 1.28) | 0.138 | - | - | N/A | N/A | N/A | N/A |
| Time-to-positivity (days) | N/A | N/A | N/A | N/A | 0.91 (0.83, 0.99) | 0.029 | - | - | 0.01 (-0.07, 0.08) | 0.835 | - | - |
| Protein concentration (g/l) | -0.96 (-2.67, 0.76) | 0.264 | - | - | 0.95 (0.66, 1.34) | 0.756 | - | - | 0.06 (-0.28, 0.40) | 0.715 | - | - |

Footnotes: The following information was unavailable for patients in this cohort: chest radiograph status, smoking status. ^*^For HIV, there were too few observations to perform a regression analysis for correlates of MTB/RIF-positivity.

Table S7. Multivariate models for liquid culture time-to-positivity, Xpert MTB/RIF-positivity and IPC C_T_ values in pleural fluid.

| Characteristic | Outcome: time-to-positivity in culture-positive patients | | | | Outcome: MTB/RIF-positivity in culture-positive patients | | | | Outcome: internal positive control C_T_ value in culture-positive patients | | | |
| --- | --- | --- | --- | --- | --- | --- | --- | --- | --- | --- | --- | --- |
|  | Univariate | | Multivariate | | Univariate | | Multivariate | | Univariate | | Multivariate | |
|  | Coefficient (95% CI) | P-value | Coefficient (95% CI) | Coefficient (95% CI) | OR (95% CI) | P-value | OR (95% CI) | P-value | Coefficient (95% CI) | P-value | Coefficient (95% CI) | P-value |
| Age | 0.02 (-0.27, 0.31) | 0.874 | - | - | 0.97 (0.90, 1.05) | 0.509 | - | - | -0.01 (-0.07, 0.05) | 0.812 | - | - |
| Male gender | 3.85 (-6.88, 9.63) | 0.726 | - | - | 0.14 (0.01, 1.76) | 0.129 | - | - | -0.86 (-2.52, 0.79) | 0.282 | - | - |
| Smoker | 0.85 (-9.77, 11.46) | 0.867 | - | - | - | - | - | - | -0.41 (-2.61, 1.79) | 0.697 | - | - |
| Previous TB | -1.69 (-13.39, 10.00) | 0.760 | - | - | 2.25 (0.11, 45.7) | 0.598 | - | - | -0.83 (-3.41, 1.75) | 0.499 | - | - |
| HIV-infected | -4.1 (-14.99, 6.79) | 0.416 | - | - | 7.5 (0.46, 122.69) | 0.158 | - | - | 0.28 (-1.92, 2.48) | 0.780 | - | - |
| MTB/RIF internal positive control C_T_ value | 0.97 (-1.72, 3.67) | 0.453 | - | - | 0.60 (0.26. 1.40) | 0.238 | - | - | N/A | N/A | N/A | N/A |
| Time-to-positivity (days) | N/A | N/A | N/A | N/A | 0.93 (0.80, 1.09) | 0.359 | - | - | 0.04 (-0.07, 0.16) | 0.453 | - | - |
| Protein concentration (g/l) | -0.17 (-0.74, 0.41) | 0.541 | - | - | 0.96 (0.82, 1.12) | 0.628 | - | - | -0.05 (-0.17, 0.07) | 0.387 | - | - |

Footnotes: The following information was unavailable for patients in this cohort: chest radiograph status. ^*^For smoking status, there were too few observations to perform a regression analysis for correlates of MTB/RIF-positivity.

Table S8. Multivariate models for liquid culture time-to-positivity, Xpert MTB/RIF-positivity and IPC C_T_ values in pericardial fluid.

| Characteristic | Outcome: time-to-positivity in culture-positive patients | | | | Outcome: MTB/RIF-positivity in culture-positive patients | | | | Outcome: internal positive control C_T_ value in culture-positive patients | | | |
| --- | --- | --- | --- | --- | --- | --- | --- | --- | --- | --- | --- | --- |
|  | Univariate | | Multivariate | | Univariate | | Multivariate | | Univariate | | Multivariate | |
|  | Coefficient (95% CI) | P-value | Coefficient (95% CI) | Coefficient (95% CI) | OR (95% CI) | P-value | OR (95% CI) | P-value | Coefficient (95% CI) | P-value | Coefficient (95% CI) | P-value |
| Age | -0.05 (-0.32, 0.22) | 0.700 | - | - | 0.97 (0.93, 1.02) | 0.297 | - | - | -0.04 (-0.09. 0.01) | 0.089 | - | - |
| Male gender | 4.70 (-2.30, 11.71) | 0.183 | - | - | 1.20 (0.31, 3.91) | 0.888 | - | - | 0.89 (-0.36, 2.14) | 0.157 | - | - |
| Previous TB | 5.26 (-1.75, 12.29) | 0.137 | - | - | 1.75 (0.44, 6.88) | 0.423 | - | - | 0.76 (-0.51, 2.04) | 0.234 | - | - |
| HIV-infected | -0.46 (-7.95, 7.03) | 0.903 | - | - | 7.20 (1.61, 32.28) | 0.010 | - | - | 1.07 (-0.23, 2.37) | 0.106 | - | - |
| MTB/RIF internal positive control C_T_ value | -0.07 (-1.77, 1.63) | 0.933 | - | - | 1.08 (0.79, 1.48) | 0.615 | - | - | N/A | N/A | N/A | N/A |
| Time-to-positivity (days) | N/A | N/A | N/A | N/A | 0.96 (0.90, 1.01) | 0.127 | - | - | 0.00 (-0.05, 0.05) | 0.933 | - | - |
| Protein concentration (g/l) | 0.11 (-0.17, 0.41) | 0.427 | - | - | 1.01 (0.96, 1.07) | 0.551 | - | - | 0.02 (-0.04, 0.07) | 0.543 | - | - |

Footnotes: The following information was unavailable for patients in this cohort: chest radiograph status, smoking status

Table S9. Multivariate models for liquid culture time-to-positivity, Xpert MTB/RIF-positivity and IPC C_T_ values in extrapulmonary specimens (pleural fluid, pericardial fluid, and CSF).

| Characteristic | Outcome: time-to-positivity in culture-positive patients | | | | Outcome: MTB/RIF-positivity in culture-positive patients | | | | Outcome: internal positive control C_T_ value in culture-positive patients | | | |
| --- | --- | --- | --- | --- | --- | --- | --- | --- | --- | --- | --- | --- |
|  | Univariate | | Multivariate | | Univariate | | Multivariate | | Univariate | | Multivariate | |
|  | Coefficient (95% CI) | P-value | Coefficient (95% CI) | Coefficient (95% CI) | OR (95% CI) | P-value | OR (95% CI) | P-value | Coefficient (95% CI) | P-value | Coefficient (95% CI) | P-value |
| Age | -0.07 (-0.24, 0.10) | 0.438 | - | - | -0.02 (-0.05, 0.02) | 0.395 | - | - | -0.03 (-0.07, -0.01) | 0.075 | -0.03 (-0.06, -0.00) | 0.076 |
| Male gender | 0.11 (-3.96, 4.18) | 0.957 | - | - | -0.13 (-0.94, 0.67) | 0.748 | - | - | -0.25 (-1.10, 0.59) | 0.553 | - | - |
| Previous TB | 4.11 (-0.50, 8.72) | 0.080 | - | - | 0.50 (-0.46, 1.45) | 0.308 | - | - | -0.02 (-0.98, 0.94) | 0.966 | - | - |
| HIV-infected | 0.47 (-4.53, 5.47) | 0.853 | - | - | 1.94 (0.75, 3.12) | 0.001 | 2.81 (1.75, 3.88) | <0.001 | 0.32 (-0.65, 1.31) | 0.505 | - | - |
| MTB/RIF internal positive control C_T_ value | 0.44 (-0.72, 1.59) | 0.452 | - | - | -0.19 (-0.42, 0.05) | 0.122 | - | - | N/A | N/A | N/A | N/A |
| Time-to-positivity (days) | N/A | N/A | N/A | N/A | -0.07 (-0.11, -0.02) | 0.004 | -0.09 (-0.11, -0.06) | <0.001 | 0.02 (-0.03, 0.07) | 0.452 | - | - |
| Specimen type (compared to pleural fluid) | | | | | | | | | | | | |
| Pericardial fluid | 1.17 (-4.82, 7.16) | 0.699 | - | - | -0.10 (-0.88, 0.67) | 0.795 | 1.93 (0.68, 3.18) | 0.002 | 0.15 (-1.03, 1.34) | 0.798 | 0.43 (-0.78, 1.63) | 0.480 |
| CSF | -1.72 (-6.18, 2.72) | 0.422 | - | - | 1.22 (0.66, 1.78) | <0.001 | 1.63 (0.97, 2.29) | <0.001 | -1.31 (-2.23, -0.38) | 0.006 | -1.41 (-2.08, -0.21) | 0.017 |

Table S10. Multivariable adjustments for Xpert MTB/RIF-positivity and IPC C_T_ values in urine.

| Characteristic | Outcome: MTB/RIF-positivity | | | | Outcome: internal positive control C_T_ value | | | |
| --- | --- | --- | --- | --- | --- | --- | --- | --- |
|  | Univariate | | Multivariate | | Univariate | | Multivariate | |
|  | OR (95% CI) | Coefficient (95% CI) | OR (95% CI) | P-value | Coefficient (95% CI) | P-value | Coefficient (95% CI) | P-value |
| Age | 0.99 (0.93, 1.06) | 0.913 | - | - | -0.06 (-0.16, 0.3) | 0.159 | - | - |
| Male gender | 0.38 (0.14, 1.07) | 0.068 | 0.49 (0.24, 1.00) | 0.051 | 0.21 (-0.6, 1.02) | 0.606 | - | - |
| Smoker | 1.35 (0.62, 2.93) | 0.448 | - | - | -0.07 (-1.02, 0.88) | 0.876 | - | - |
| Previous TB | 0.50 (0.24, 1.01) | 0.054 | 0.50 (0.24, 1.01) | 0.054 | 0.24 (-0.69, 1.17) | 0.605 | - | - |
| Chest radiograph compatible with active TB | 1.67 (0.58, 4.83) | 0.345 | - | - | -0.60 (-2.01, 0.71) | 0.396 | - | - |
| CD4 count | 1.00 (1.00-1.00) | 0.827 | - | - | 0.00 (0.00-0.01) | 0.300 | - | - |
| MTB/RIF internal positive control C_T_ value^*^ | - | - | - | - | N/A | N/A | N/A | N/A |
| Protein concentration (g/l) | 1.75 (0.69, 4.24) | 0.237 | - | - | 0.81 (-0.76, 1.70) | 0.072 | - | - |

Footnotes: All patients in this cohort were HIV-infected and none had culture-positive urine. ^*^Internal positive control C_T_ values were not recorded for MTB/RIF-negative specimens.
